# Reference left atrial dimensions and volumes by steady state free precession cardiovascular magnetic resonance

**DOI:** 10.1186/1532-429X-12-65

**Published:** 2010-11-11

**Authors:** Alicia M Maceira, Juan Cosín-Sales, Michael Roughton, Sanjay K Prasad, Dudley J Pennell

**Affiliations:** 1 Cardiac Imaging Unit, ERESA, Valencia, Spain; 2 Dept. of Cardiology, Hospital Arnau de Vilanova, Valencia, Spain; 3 Medical Statistics Department, Royal Brompton Hospital, London, UK; 4 Cardiovascular Magnetic Resonance Unit, Royal Brompton Hospital, London, UK

## Abstract

**Background:**

Left atrial (LA) size is related to cardiovascular morbidity and mortality. Cardiovascular magnetic resonance (CMR) provides high quality images of the left atrium with high temporal resolution steady state free precession (SSFP) cine sequences. We used SSFP cines to define normal ranges for LA volumes and dimensions relative to gender, age and body surface area (BSA), and examine the relative value of 2D atrial imaging techniques in patients.

For definition of normal ranges of LA volume we studied 120 healthy subjects after careful exclusion of cardiovascular abnormality (60 men, 60 women; 20 subjects per age decile from 20 to 80 years). Data were generated from 3-dimensional modeling, including tracking of the atrioventricular ring motion and time-volume curves analysis. For definition of the best 2D images-derived predictors of LA enlargement, we studied 120 patients (60 men, 60 women; age range 20 to 80 years) with a clinical indication for CMR.

**Results:**

In the healthy subjects, age was associated with LA 4-chamber transverse and 3-chamber anteroposterior diameters, but not with LA volume. Gender was an independent predictor of most absolute LA dimensions and volume, but following normalization to BSA, some associations became non-significant. CMR normal ranges were modeled and are tabled for clinical use with normalization, where appropriate, for BSA and gender and display of parameter variation with age. The best 2D predictors of LA volume were the 2-chamber area and 3-chamber area (both r = 0.90, p < 0.001).

**Conclusions:**

These CMR data show that LA dimensions and volume in healthy, individuals vary significantly by BSA, with lesser effects of age and gender.

## Background

Left atrial (LA) size represents the integration of LV diastolic performance over time and is considered a reliable indicator of the duration and severity of diastolic dysfunction [1], regardless of whatever loading conditions are present at the time of the examination. It provides significant prognostic information both in the general population and in patients with heart disease including heart failure [2-4], acute myocardial infarction [5-8], cardiomyopathy [9,10], and mitral regurgitation [11]. LA enlargement is commonly found in hypertensive heart disease [12,13] and it is a risk factor for atrial fibrillation and stroke, especially in men [14,15], and for atrial fibrillation recurrence following therapy [16,17]. In the clinical setting, LA diameters and areas are usually measured, though LA volume is a more robust marker of cardiovascular events [18]. Cardiovascular magnetic resonance (CMR) has been applied for the measurement of left and right ventricular volumes, systolic function and mass for many years in the clinical arena, with standardized methods of short axis multi-slice acquisition [19]. The excellent accuracy and reproducibility of CMR is well established [20], making it a gold standard technique for measurement of ventricular dimensions and function, for which reference ranges have been established from the Steady State Free Precession (SSFP) technique [21,22]. SSFP yields excellent blood-endocardium and epicardium-fat contrast, higher acquisition speed, and the ability to greatly improve the temporal resolution of the cines without loss of image quality [23]. However, atrial dimensions have not been extensively studied with CMR, and there is limited data on the influences of age, gender and body surface area (BSA) on atrial dimensions. Therefore, the aim of this study was to establish SSFP based reference values in normal subjects for LA dimensions normalized, when necessary, for independent influences such as gender, body surface area and age. It was also our aim to determine the best predictors of LA enlargement among 1D and 2D parameters. Finally, we produced an equation for simplified estimation of LA volume from LA diameters and areas easily obtained in a clinical setting.

## Methods

### Patients

For definition of normal ranges of LA dimensions we studied 120 subjects, with 10 men and 10 women in each of 6 age deciles from 20 to 80 years, these subjects having been reported elsewhere in a study of ventricular volumes [21] in which, importantly, their diastolic filling parameters by filling rate were shown to be normal. All subjects were normotensive (hypertension defined as systolic blood pressure ≥ 140 mmHg and/or diastolic blood pressure ≥ 90 mmHg), completely asymptomatic, with no known risk factors or history of cardiac disease, and normal physical examination and electrocardiogram (ECG). Also measured were the height, weight, blood pressure, total cholesterol, HDL and B-natriuretic peptide. BSA was calculated according to the Mosteller formula [24]. With this information, the coronary artery disease risk over 10 years was calculated [25]. BNP levels were 2.5 ± 2.1 pg/mL (range 0.5 - 12.0), and all were in the normal range (<100 pg/mL) [26]. Therefore, as far as it was possible to ascertain with conventional noninvasive techniques, all the apparently healthy subjects had a normal cardiovascular system. The baseline characteristics of these healthy subjects have been published elsewhere [21]. For definition of the best 2D images-derived predictors of LA enlargement, a group of 120 patients (60 men and 60 women) who were referred to CMR for clinical reasons, and who agreed to participate in the study, were also studied. The main reasons for referral to CMR are summarized in table 1. The study was approved by the institutional ethics committee, and all subjects gave written informed consent.

**Table 1:** Baseline characteristics of the healthy subjects and the patient group (mean ± SD)

	Healthy subjects	Patients
N	120	120

Males	50%	50%

Age [yr]	49 ± 17	65 ± 12

Height [m]	171 ± 9	163 ± 9

Weight [kg]	72 ± 13	77 ± 13

Body surface area [m^2^]	1.83 ± 0.18	1.83 ± 0.18

Body mass index [kg/m^2^]	24 ± 4	29 ± 5

Heart Rate [bpm]	66 ± 10	70 ± 14

Systolic blood pressure [mmHg]	124 ± 12	141 ± 26

Diastolic blood pressure [mmHg]	73 ± 7	77 ± 15

Referral (n)		

Ischemic heart disease	-	47

Coronary risk factors	-	35

Hypertensive heart disease	-	13

Valvular heart disease	-	12

Dilated cardiomyopathy	-	4

Restrictive cardiomyopathy	-	2

Congenital heart disease	-	2

Myocarditis	-	2

Hypertrophic cardiomyopathy	-	1

Arrhythmogenic right ventricular cardiomyopathy	-	1

Pericardial disease	-	1

### CMR

CMR was performed with 1.5T scanners (Siemens Sonata and Avanto) using front and back surface coils and retrospective ECG triggering for capture of the entire cardiac cycle including diastole. All CMR scans were acquired by the same operator. SSFP end-expiratory breath-hold cines were acquired in the 2, 4 and 3 chamber views, with subsequent contiguous short-axis cines from the atrioventricular (AV) ring to the base of the atria. Slice thickness was 5 mm with no gap between slices. The temporal resolution was 21 ± 1 ms. Sequence parameters included repetition time/echo time of 3.2/1.6 ms, in-plane pixel size of 2.1 × 1.3 mm, flip angle 60°, and acquisition time of typically 18 heartbeats.

### CMR analysis

**Figure 1:**
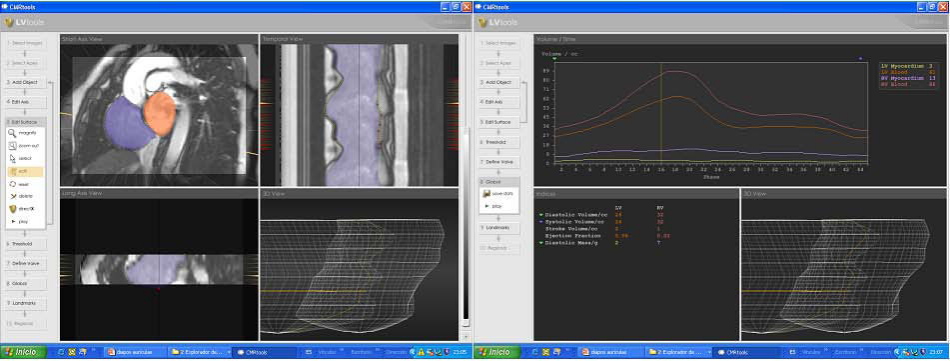
**CMR analysis of atrial volumes**. Atrial endocardial borders were delineated in all planes in all cardiac phases. The systolic descent and twist of the mitral valve was calculated from tracking of the valve motion on the long axis cines.

**Figure 2:**
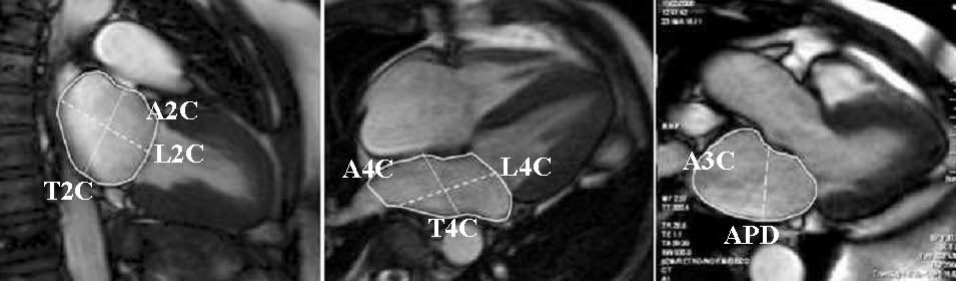
**Measurement of LA parameters in the end-systolic phase of the cardiac cycle**. Areas were measured in the 2, 4 and 3 chamber views (A2C, A4C, A3C). Longitudinal and transverse diameters were measured in the 2 and 4 chamber views (L2C, T2C, L4C, T4C) and the anteroposterior diameter in the 3 chamber view (APD)

Analysis was performed with a personal computer and semi-automated software (CMRTools, Cardiovascular Imaging Solutions, London, UK). In all subjects (healthy controls and patients) LA maximum volume was measured as well as end-systolic diameters and areas. Atrial volume analysis included 2 steps: First, delineation of the atrial endocardial borders, including atrial appendage, in all planes in all cardiac phases. Second, the systolic descent and twist of the mitral valve was calculated from tracking of the valve motion on the long axis cines, and used to correct for increase in atrial volume due to AV ring descent (figure 1). In the analysis we included the atrial appendage and excluded the pulmonary veins. All atrial parameters derived from 2D images were measured in the end-systolic phases of the corresponding cine sequences in order to obtain maximum diameters and areas. LA areas were planimetered in the 4 chamber view, 2 chamber view and in the 3 chamber view or LVOT view. Longitudinal and transverse diameters were measured in the 4 and 2 chamber views. LA anteroposterior diameter was also measured in the 3 chamber view (figure 2).

### Statistical analysis

All atrial parameters were found to satisfy a normal distribution using the Kolmogorov-Smirnov test and summary data for these variables are therefore presented as mean ± SD. The interobserver variability was measured in a subset of 20 subjects for all variables. Simple linear regression was used to model the data and to construct reference ranges as mean and 95% confidence intervals. Two-way ANOVA was used to analyze variations in parameters due to age and gender. P values < 0.05 were considered significant. In the patient group correlations of 1D and 2D parameters with LA volume were assessed with the Pearson's coefficient. Logistic regression analysis was used to define the best predictors of LA enlargement among 1D and 2D parameters. Linear regression analysis was used to predict LA volume.

## Results

### Baseline characteristics and summary results

Table 1 summarizes the baseline characteristics of the healthy subjects included for defining normal reference values. Normal reference values with differentiation into males, females and all subjects, without age breakdown, and sub-division into absolute and body surface area normalized values are shown for the left atrium (table 2), which have application in studies of unsorted individuals. Parameters that showed differences with age are also depicted, with age breakdown, in table 3. The interobserver variability was 2.9% and 3.2% for longitudinal diameters in the 2 and 4 chamber views, 3.4% and 3.8% for transverse diameters in the 2 and 4 chamber views, 3.5% for the anteroposterior diameter, 4.4%, 4.7% and 3.9% for areas in the 2, 4 and 3 chamber views and 5.7% for LA volume.

**Table 2:** Healthy subjects-Left atrial summary data for all ages (mean, 95% confidence interval)

	All	Males	Females
Volume [mL] SD 14.9 *	73 (44, 102)	77 (48, 107)	68 (42, 95)
Volume/BSA [mL/m^2^] SD 6.7	40 (27, 53)	39 (26, 53)	40 (27, 52)
Area - 4ch [cm^2^] SD 3.7 *	21 (14, 28)	22 (14, 30)	20 (14, 27)
Area/BSA - 4ch [cm^2^/m^2^] SD 1.8 *	12 (8, 15)	11 (7, 15)	12 (8, 15)
Longitudinal diameter - 4ch[cm] SD 0.7 *	5.7 (4.3, 7.0)	5.9 (4.5, 7.2)	5.5 (4.1, 6.9)
Longitudinal diameter/BSA - 4ch[cm/m^2^] SD 0.4 *	3.1 (2.3, 3.9)	3.0 (2.3, 3.7)	3.2 (2.4, 4.1)
Transverse diameter - 4ch [cm] SD 0.5	4.1 (3.0, 5.1)	4.1 (3.0, 5.2)	4.1 (3.0, 5.1)
Transverse diameter/BSA - 4ch [cm/m^2^] SD 0.3 *	2.2 (1.6, 2.8)	2.1 (1.5, 2.8)	2.4 (1.8, 3.0)
Area - 2ch [cm^2^] SD 4.7 *	20 (11, 29)	21 (12, 31)	19 (10, 28)
Area/BSA - 2ch [cm^2^/m^2^] SD 2.4	11 (6, 16)	11 (6, 15)	11 (6, 16)
Longitudinal diameter - 2ch[cm] SD 0.7 *	4.9 (3.4, 6.3)	5.0 (3.6, 6.4)	4.6 (3.1, 6.0)
Longitudinal diameter/BSA - 2ch[cm/m^2^] SD 0.4	2.6 (1.9, 3.2)	2.5 (1.8, 3.2)	2.7 (1.2, 4.1)
Transverse diameter - 2ch [cm] SD 0.5 †	4.6 (3.6, 5.6)	4.6 (3.7, 5.5)	4.4 (3.4, 5.5)
Transverse diameter/BSA - 2ch [cm/m^2^] SD 0.2 * †	2.5 (2.0, 2.9)	2.3 (1.9, 2.8)	2.6 (1.5, 3.6)
Area - 3ch [cm^2^] SD 3.6 * †	18 (11, 25)	19 (13, 26)	17 (11, 24)
Area/BSA - 3ch [cm^2^/m^2^] SD 1.8 †	10 (6, 13)	10 (6, 13)	10 (7, 13)
AP diameter - 3ch [cm] SD 0.5 †	3.2 (2.2, 4.2)	3.3 (2.3, 4.2)	3.1 (2.1, 4.1)
AP diameter/BSA - 3ch [cm/m^2^] SD 0.3 * †	1.7 (1.2, 2.3)	1.7 (0.7, 2.6)	1.8 (1.3, 2.3)

* Significant differences (p < 0.05) between males and females on multivariable analysis

† Significant differences (p < 0.05) among age groups on multivariable analysis

BSA - body surface area; 4ch - 4-chamber view; 2ch - 2-chamber view; 3ch - 3-chamber view or LVOT view; AP - anteroposterior

**Table 3:** Healthy subjects-Left atrial parameters significantly influenced by age on multivariable analysis (mean, 95% confidence interval)

	20-29 years	30-39 years	40-49 years	50-59 years	60-69 years	70-79 years
	**All**					

Transverse diameter - 2ch [cm] SD 0.5	4.8 (3.9, 5.8)	4.7 (3.8, 5.7)	4.7 (3.7, 5.6)	4.6 (3.6, 5.5)	4.5 (3.5, 5.4)	4.4 (3.4, 5.4)

AP diameter-3ch [cm] SD 0.6	4.9 (3.8, 6.0)	4.8 (3.7, 5.9)	4.6 (3.5, 5.7)	4.5 (3.4, 5.6)	4.3 (3.2, 5.5)	4.2 (3.1, 5.3)

Area/BSA-3ch [cm] SD 1.8	9 (5, 12)	9 (6, 13)	10 (6, 13)	10 (7, 14)	10 (7, 14)	11 (7, 14)

	**Males**					

Transverse diameter/BSA-2ch [cm] SD 0.2	2.5 (2.0, 2.9)	2.4 (1.9, 2.9)	2.4 (1.9, 2.8)	2.3 (1.8, 2.8)	2.2 (1.8, 2.7)	2.2 (1.7, 2.7)

AP diameter/BSA-3ch [cm] SD 0.3	2.7 (2.0, 3.4)	2.6 (1.9, 3.2)	2.5 (1.8, 3.1)	2.3 (1.7, 3.0)	2.2 (1.5, 2.9)	2.1 (1.4, 2.8)

Area-3ch [cm] SD 3.8	17 (11, 24)	18 (11, 25)	19 (12, 25)	20 (13, 26)	21 (14, 27)	21 (15, 28)

	**Females**					

Transverse diameter/BSA-2ch [cm] SD 0.3	2.8 (2.2, 3.3)	2.7 (1.6, 3.7)	2.6 (1.6, 3.7)	2.6 (1.5, 3.6)	2.5 (1.4, 3.6)	2.4 (1.4, 3.5)

AP diameter/BSA-3ch [cm] SD 0.3	2.8 (2.3, 3.4)	2.8 (2.2, 3.3)	2.7 (2.1, 3.3)	2.6 (2.0, 3.2)	2.5 (1.9, 3.1)	2.4 (1.9, 3.0)

Area-3ch [cm] SD 3.4	16 (9, 22)	16 (10, 23)	17 (10, 24)	18 (11, 24)	18 (12, 25)	19 (12, 26)

AP - anteroposterior; 4ch - 4-chamber view; 2ch - 2-chamber view; 3ch - 3 chamber view; BSA - body surface area; SD - standard deviation

### Influence of body surface area on atrial parameters

BSA was significantly higher in males than in females (p < 0.001). On multivariate analysis, BSA was found to have significant independent influence on all LA parameters except for the longitudinal and transverse diameters in the 4-chamber view.

### Influence of age on atrial parameters

No significant increase in LA volume with age was observed. On univariable analysis there was a significant decrease in absolute and normalized transverse diameters (measured in the 2-chamber view) (p = 0.038, p = 0.008, respectively), and in absolute and normalized anteroposterior diameters (both p < 0.001) with increasing age. On multivariable analysis, age was an independent predictor of absolute and normalized transverse diameters (measured in the 2-chamber view) (p = 0.022 and p = 0.004, respectively) and of absolute and normalized anteroposterior diameters and areas (measured in the 3-chamber view) (diameters p = 0.001 and p = 0.001, areas p = 0.005 and p = 0.006). Age was not an independent predictor of LA volume. Variables with significant differences according to age in the multivariable analysis are depicted with age breakdown in table 3.

### Influence of gender on atrial parameters

All absolute LA volume, diameters and areas were significantly larger in males (all p < 0.05) except transverse diameters (2-chamber and 4-chamber views) and anteroposterior diameter, which did not show significant differences. When parameters were normalized to BSA, only longitudinal (4-chamber view), transverse (2 and 4-chamber views) and anteroposterior diameters showed differences, being all of them higher in females (all p < 0.01). On multivariable analysis, gender had significant independent influence on absolute longitudinal diameters (2-chamber and 4-chamber), areas (2-chamber, 3-chamber, 4-chamber views) and volume, and on normalized longitudinal (4-chamber), transverse (2-chamber and 4-chamber) and anteroposterior diameters and area (4-chamber view) (all p < 0.01).

### Predictors of atrial enlargement

Table 1 also depicts the baseline characteristics of the patient group. As expected, in the patient group atrial volumes showed a significant dispersion. Thus, LA volume index (LAVi) ranged from 20 to 218 mL/m^2 ^(mean ± SD = 63 ± 39 mL/m^2^). According to our own normal reference values reported in table 2, 70 patients (38 males, 32 females) had LA enlargement (LAVi >53 mL/m^2^). We aimed to determine the best independent predictors of LA enlargement, for which multivariable logistic regression analysis with forward selection was performed for normalized 1D and 2D parameters. In order to simplify the results, these parameters were included as categorical dichotomous variables (above or below the upper limit of normal for all subjects for each parameter). This analysis showed that the best predictors of LA enlargement were normalized area and normalized transverse diameter in the 4-chamber view (normalized area: RR = 2.22, p = 0.001, 95%CI = 1.60, 3.09; normalized transverse diameter: RR = 1.23, p = 0.023, 95%CI = 1.03, 1.47) (table 4).

**Table 4:** Predictors of left atrial enlargement according to normalized LAV

Univariable analysis: Measurements	RR	95%CI	P value	Chi square
Longitudinal diameter-4ch (> 7 cm)	1.862	1.562, 2.220	< 0.001	NA
Transverse diameter-4ch (> 5.1 cm)	3.263	1.811, 5.880	< 0.001	15.5
Area-4ch (> 28 cm^2^)	2.199	1.674, 2.889	< 0.001	32.1
Longitudinal diameter-2ch (> 6.3 cm)	2.074	1.543, 2.787	< 0.001	23.4
Transverse diameter-2ch (> 5.6 cm)	1.911	1.481, 2.466	< 0.001	24.8
Area-2ch (> 29 cm^2^)	1.833	1.419, 2.368	< 0.001	21.6
AP diameter-3ch (> 4.2 cm)	1.741	1.298, 2.337	< 0.001	13.7
Area-3ch (> 25 cm^2^)	2.205	1.648, 2.951	< 0.001	28.3
Longitudinal diameter/BSA-4ch (> 3.9 cm/m^2^)	1.980	1.631, 2.404	< 0.001	NA
Transverse diameter/BSA-4ch (> 2.8 cm/m^2^)	1.657	1.280, 2.145	< 0.001	14.7
Area/BSA-4ch (> 15 cm^2^/m^2^)	2.413	1.776, 3.279	< 0.001	31.7
Longitudinal diameter/BSA-2ch (> 3.2 cm/m^2^)	2.168	1.643, 2.861	< 0.001	29.9
Transverse diameter/BSA-2ch (> 2.9 cm/m^2^)	1.646	1.195, 2.268	0.002	9.3
Area/BSA-2ch (> 16 cm^2^/m^2^)	2.205	1.659, 2.932	< 0.001	19.6
AP diameter/BSA-3ch (> 2.3 cm/m^2^)	2.013	1.461, 2.774	< 0.001	18.3
Area/BSA-3ch (> 13 cm^2^/m^2^)	2.255	1,675, 3.037	< 0.001	28.7

Multivariable analysis: Normalized measurements				

Area/BSA-4ch (> 15 cm^2^/m^2^)	2.221	1.595, 3.094	< 0.001	46.2
Transverse diameter/BSA-4ch (> 2.8 cm/m^2^)	1.228	1.028, 1.468	0,023	

The upper limit of normal for each parameter is shown in brackets according to table 2.

AP - anteroposterior; 4ch - 4-chamber view; 2ch - 2-chamber view; 3ch - 3 chamber view; BSA - body surface area

### Estimation of LA volume from 2D based dimensions in the patient group

All 1D and 2D parameters correlated significantly with LA volume. The best correlations were found for areas measured in the 2-chamber (r = 0.90, p < 0.001) and 3-chamber views (r = 0.90, p < 0.001) (Table 5). Finally, linear regression analysis was used to estimate LA volume (Table 6). All absolute 1D and 2D measurements were included in multiple linear regression analysis and the equation obtained for LA volume (r^2 ^= 0.88) was:

**Table 5:** Correlations of 1D and 2D parameters with left atrial volume (Pearson's coefficient)

Parameter	Pearson's coefficient	P
Longitudinal diameter -4ch	0.769	0.000
Transverse diameter -4ch	0.748	0.000
Area -4ch	0.870	0.000
Longitudinal diameter -2ch	0.777	0.000
Transverse diameter -2ch	0.847	0.000
Area -2ch	0.904	0.000
AP diameter -3ch	0.685	0.000
Area -3ch	0.903	0.000

AP - anteroposterior; 4ch - 4-chamber view; 2ch - 2-chamber view; 3ch - 3 chamber view

**Table 6:** Predictors of absolute left atrial volume

Univariable analysis: Measurements	Coeff	95%CI	P value	R squared
Longitudinal diameter -4ch [cm]	2.979	2,551,3.406	< 0.001	0.614
Transverse diameter -4ch [cm]	3.435	2.924,3.945	< 0.001	0.597
Area -4ch [cm^2^]	3.643	3.230,4.056	< 0.001	0.719
Longitudinal diameter -2ch [cm]	3.080	2.599,3.561	< 0.001	0.604
Transverse diameter -2ch [cm]	3.200	2.774,3.626	< 0.001	0.678
Area -2ch [cm^2^]	3.325	2.941,3.710	< 0.001	0.711
AP diameter -3ch [cm]	2.688	2.135,3.240	< 0.001	0.436
Area -3ch [cm^2^]	3.925	3.499,4.352	< 0.001	0.753
Longitudinal diameter/BSA -4ch [cm/m^2^]	3.992	3.159,4.824	< 0.001	0.428
Transverse diameter/BSA -4ch [cm/m^2^]	4.574	3.597,5.551	< 0.001	0.417
Area/BSA -4ch [cm^2^/m^2^]	5.641	4.821,6.462	< 0.001	0.608
Longitudinal diameter/BSA -2ch [cm/m^2^]	4.059	3.114,5.004	< 0.001	0.405
Transverse diameter/BSA -2ch [cm/m^2^]	4.408	3.524,5.291	< 0.001	0.480
Area/BSA -2ch [cm^2^/m^2^]	5.413	4.667,6.159	< 0.001	0.633
AP diameter/BSA -3ch [cm/m^2^]	3.754	2.770,4.739	< 0.001	0.320
Area/BSA -3ch [cm^2^/m^2^]	6.502	5.649,7.356	< 0.001	0.676

**Multivariable analysis: Absolute measurements**				

Area -3ch [cm^2^]	1.896	0.957, 2.836	< 0.001	0.902
Area -4ch [cm^2^]	1.119	0.154, 2.085	0.024	
AP diameter -3ch [cm]	-1.653	-2.492, -0.814	< 0.001	
Transverse diameter -2ch [cm]	1.080	0.463, 1.696	0.001	
Transverse diameter -4ch [cm]	0.925	0.147, 1.703	0.02	
Constant	3.314	3.052, 3.577		

AP - anteroposterior; 4ch - 4-chamber view; 2ch - 2-chamber view; 3ch - 3 chamber view; BSA - body surface area

LAV=3.31+[1.9*A3]+[1.1*A4]+[1.1*TD2]+[0.9*TD4]−[1.7*APD]

Where LAV is LA volume (mL), A3 is area in the three-chamber view, A4 is area in the four-chamber view (both in cm2), TD2 is transverse diameter in the two-chamber view, TD4 is transverse diameter in the four-chamber view and APD is anteroposterior diameter (all in cm).

**Figure 3:**
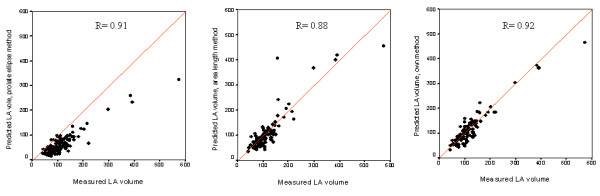
**Graphs showing agreement of the prolate ellipse and area-length methods as well as the new method**.

This method was compared with the traditional echocardiography derived area-length and prolate ellipse equations [27]. Correlation coefficients with real LA volume were r = 0.91 for the prolate ellipse method, r = 0.88 for the area-length method and r = 0.92 for our method (figure 3). The three methods underestimated real volume, with a mean difference of 50 ± 32 mL for the prolate ellipse method, 22 ± 30 mL for the area-length method and 17 ± 16 mL for our method.

## Discussion

This current study provides normal reference ranges for atrial dimensions using modern CMR acquisition techniques and analysis for a healthy population which has been very well characterized for the absence of hypertension, significant coronary disease and heart failure. CMR is now considered a gold standard clinical technique to measure atrial and ventricular volumes and function, so these data have significant clinical utility. The tables of results include all 1D and 2D parameters as well as LA volume and are divided into males/females or all subjects, and in age deciles, when appropriate, or all ages, in order to have applicability for comparison with any other future research data set. For all ages and genders, a volume of 102 mL (53 mL/m2) was obtained as the upper limit of normality. With regard to areas, the upper limits of normality were 28 cm^2 ^(15 cm^2^/m^2^) in the four chamber view, 29 cm^2 ^(16 cm^2^/m^2^) in the 2-chamber view and 25 cm^2 ^(13 cm^2^/m^2^) in the 3-chamber view. The upper limits of normality for diameters in the 4-chamber and 2-chamber views were longitudinal 7.0 cm (3.9 cm/m^2^) and 6.3 cm (3.2 cm/m^2^), transverse 5.1 cm (2.8 cm/m^2^) and 5.6 cm (2.9 cm/m^2^) respectively, and an anteroposterior diameter of 4.2 cm (2.3 cm/m^2^) in the 3-chamber view.

Other authors have published reference ranges for atrial dimensions with CMR but we have not found any other study in which all 1D, 2D and 3D parameters were measured with CMR. Several studies in the past addressed LA dimensions with spin-echo sequences that are not comparable with the current steady state free precession sequences that we used. Hudsmith et al [28], using the biplane area-length method, established values of volume similar to ours, 97 ± 27 mL for all subjects, 103 ± 30 mL for males and 89 ± 21 mL for females, with differences probably due to the need for geometric assumptions with that method. Sievers et al [29], published reference diameters for the 2, 4 and 3-chamber views slightly lower than ours, probably due to the fact that they used prospective triggering, which yields lower values, as reported by the same authors [30]. Anderson et al [31] published that a LA area < 24 cm^2 ^and a depth < 5.8 cm included the upper 95^th ^percentile of the normal range. Therkelsen et al [32] obtained higher values in a small group of 19 volunteers, with a mean LA volume of 62 mL/m^2^.

We observed that nearly all non-indexed 1D, 2D and 3D parameters were significantly higher in males, except for transverse diameters in the 2 and 4 chamber views and the anteroposterior diameter in the 3-chamber view, while these differences disappeared in most normalized parameters, though some of them even turned out to be higher in females. Hudsmith et al [28] also reported higher absolute LA volumes in males with similar ejection fraction between the two genders. Similarly, Sievers et al observed higher diameters in the 2 and 4-chamber views in males with no differences in the anteroposterior diameter [29].

With respect to age, we found no differences in LA volume with increasing age in this group of healthy, normotensive individuals, and only six 1D and 2D parameters showed significant differences: absolute and normalized transverse diameter in the 2-chamber view and absolute and normalized anteroposterior diameter and area the 3-chamber view. This finding is in accord with previous reports [33,34] and could indicate that age causes a certain degree of atrial remodeling without significant atrial dilatation. The LA is exposed to left ventricular diastolic pressure and, because of its thin walls, tends to dilate when pressure increases. Our healthy population was normotensive, with no signs of coronary artery disease, cardiomyopathy - the main causes of significant diastolic dysfunction - or valvular heart disease. Thus, our aged healthy volunteers were very likely to have just a mild degree of diastolic dysfunction, the so-called stage 1, with no increased filling pressures and no concomitant LA dilatation. Diastolic function parameters derived from ventricular time-volume curves in this healthy population have been published elsewhere [21]. Concordantly, Thomas et al [35] measured LA volume by 3D echocardiography in 92 healthy subjects and found that normal aging does not increase LA size. Sievers et al [29] compared volunteers less than 50 years with those over that age and observed no significant differences. Germans et al [36], comparing 19 healthy subjects between 20-40 years versus another 19 between 40-65 years, found that maximum LA volume tended to be larger in the older group, but this difference did not reach statistical significance. They obtained values for LA volume slightly higher than ours, approximately 50 ± 7 mL/m^2^. Of note, they excluded subjects with hypertension defined as systolic blood pressure ≥160 mmHg and/or diastolic blood pressure ≥90 mmHg, which means that subjects with mild hypertension could have been included who had higher LA volumes.

### Comparison with echocardiographic studies and other imaging techniques

Since CMR does not require for geometric assumptions to measure LA volume and allows inclusion of the LA appendage into the volume measurement, LA volume measured by retrogated CMR is said to be larger than the reference values obtained with 1D and 2D echocardiography, mainly by the area-length, Simpson's and prolate ellipsoid methods. Ukino et al [37] reported reference LA volumes that ranged from 39 ± 14 mL/m^2 ^by the area-length method to 32 ± 14 mL/m^2 ^by the prolate-ellipsoid method, smaller than our values. Currently, 3D echo is the preferred echocardiographic technique for measuring LA volume because of its higher accuracy. Keller et al [38] showed that 3D echo had the highest correlation and lowest bias compared to CMR, with a mild underestimation of volume of 5.3 mL. Artang et al [39] have recently found a systematic underestimation of LA volumes with 3D echo when compared to CMR, of 15-20 mL, that could be due to the higher spatial resolution of CMR which permits more accurate border detection and better delineation of volumes within the trabeculae. Also, the lower temporal resolution of 3D echo may account for these differences.

Cardiac computed tomography (CCT) has also been used to measure LA volume, with published reference values higher than the ones reported in our study. Lin et al [40] measured LA volume with CCT in 103 healthy normotensive volunteers and obtained a reference value of 102 ± 48 mL. Likewise, Mahabadi et al [41] studied 96 patients in whom mean LA volume was 90 ± 25 mL. The reason for this disparity of results might be the characteristics of the patients recruited, as the ones in the study from Mahabadi were a subset of the Rule Out Myocardial Infarction using Computer Assisted Tomography trial.

### Predictors of LA enlargement and estimators of LA volume

Though measurement of LA volume is desirable, it may be too time-consuming for daily clinical practice. Therefore, 1D and 2D parameters might be a valuable tool to assess LA size. The best independent indicators of LA enlargement in our study were an area > 15 cm^2^/m^2 ^and a transverse diameter > 2.8 cm/m^2 ^in the 4-chamber view. In the study by Anderson et al [31], an absolute LA area < 24 cm^2 ^and depth < 5.8 cm were the parameters that best distinguished normal from abnormal atria. With respect to LA volume estimators, we found that the best method included measurement of area in the 3 and 4-chamber views, transverse diameter in the 2 and 4-chamber views and the anteroposterior diameter. We correlated real volume with estimated volumes derived from this method and from the traditionally used biplane area-length and prolate ellipsoid methods. All of them correlated well but caused a significant underestimation of LA volume, with the worse accuracy found for the prolate ellipsoid method (mean difference of 50 ± 32 mL). Some studies have also compared methods of volume estimation with real volumes, with different results. Sievers et al [42] compared, in a group of 15 healthy subjects, the biplane area-length method with real volume, showing that the biplane area-length method caused a significant overestimation. An echocardiographic study by Badano et al [43] that sought to assess how many patients would be misclassified using M-mode or 2D estimates of LA size instead of real LA volume, showed that both 1D and 2D parameters were poor predictors of LA volume, especially in enlarged atria. Like the single plane area-length method, the prolate-ellipsoid method assumes an ellipsoid geometry for the LA but systematically calculates smaller volumes than the biplane methods, which stems from any error with the section of the 3 pairs of coordinates creating a large difference in the volume measurement. The biplane methods require planimetry from 2 orthogonal planes and any single error in tracing the endocardium is more forgiving because it is only 1 point among multiple points used for such tracing. Even the well-validated biplane methods have been shown to systematically underestimate LA volume when compared with MRI or CCT [44].

## Conclusions

Atrial dimensions vary mainly by body surface area, with lesser effects of gender and age. Identification particularly of early abnormality requires reference ranges which normalize for all 3 variables. These ranges are supplied with this report in both tabular and graphical form and are of significant clinical and research utility for the interpretation of CMR studies. Also, best predictors of LA enlargement are provided.

## List of abbreviations used

(LA): Left atrial; (CMR): Cardiovascular magnetic resonance; (SSFP): Steady state free precession; (AV): Atrioventricular; (LVOT): Left ventricular outflow tract.

## Competing interests

Dr Pennell is a consultant to Siemens and a director of Cardiovascular Imaging Solutions. The other authors have no conflicts to declare.

## Authors' contributions

AMM - Study design, subject scanning, data selection, data processing, writing manuscript.

JCS - Subject recruitment, data selection, data processing.

MR- Study design, statistical analysis.

SKP - Study design, subject scanning.

DJP - Study design, editing manuscript.

All authors have read and approved the final manuscript.

## References

[B1] Simek CL Feldman MD Haber HL Wu CC Jayaweera AR Kaul S Relationship between left ventricular wall thickness and left atrial size: comparison with other measures of diastolic function J Am Soc Echocardiogr 1995 8 37 47 10.1016/S0894-7317(05)80356-6 7710749

[B2] Rossi A Cicoira M Florea VG Golia G Florea ND Khan AA Murray ST Nguyen JT O'Callaghan P Anand IS Coats A Zardini P Vassanelli C Henein M Chronic heart failure with preserved left ventricular ejection fraction: diagnostic and prognostic value of left atrial size Int J Cardiol 2006 110 386 92 10.1016/j.ijcard.2005.08.049 16325283

[B3] Rossi A Cicoira M Zanolla L Sandrini R Golia G Zardini P Enriquez-Sarano M Determinants and prognostic value of left atrial volume with dilated cardiomyopathy J Am Coll Cardiol 2002 40 1425 1430 10.1016/S0735-1097(02)02305-7 12392832

[B4] Melenovsky V Borlaug BA Rosen B Hay I Ferruci L Morell CH Lakatta EG Najjar SS Kass DA Cardiovascular features of heart failure with preserved ejection fraction versus nonfailing hypertensive left ventricular hypertrophy in the urban Baltimore community: the role of atrial remodeling/dysfunction J Am Coll Cardiol 2007 49 198 207 10.1016/j.jacc.2006.08.050 17222731

[B5] Dini FL Cortigiani L Baldini U Boni A Nuti R Barsotti L Micheli G Prognostic value of left atrial enlargement in patients with idiopathic dilated cardiomyopathy and ischemic cardiomyopathy Am J Cardiol 2002 89 518 23 10.1016/S0002-9149(01)02290-1 11867034

[B6] Moller JE Hillis GS Oh JK Seward JB Reeder GS Wright RS Park SW Bailey KR Pellikka PA Left atrial volume. A powerful predictor of survival after acute myocardial infarction Circulation 2003 107 2207 12 10.1161/01.CIR.0000066318.21784.43 12695291

[B7] Beinart R Boyko V Schwammenthal E Kuperstein R Sagie A Hod H Matetzky S Behar S Eldar M Feinberg MS Long-term prognostic significance of left atrial volume in acute myocardial infarction J Am Coll Cardiol 2004 44 327 34 10.1016/j.jacc.2004.03.062 15261927

[B8] Meris A Amigoni M Uno H Thune JJ Verma A Kober L Bourqoun M McMurray JJ Velazquez EJ Maggioni AP Ghali J Arnold JM Zelenkofske S Pfeffer MA Solomon SD Left atrial remodelling in patients with myocardial infarction complicated by heart failure, left ventricular dysfunction, or both: the VALIANT Echo study Eur Heart J 2009 30 56 65 10.1093/eurheartj/ehn499 19001474

[B9] Kizer JR Bella JN Palmieri V Liu JE Best LG Lee ET Roman MJ Devereux RB Left atrial diameter as an independent predictor of first clinical cardiovascular events in middle-aged and elderly adults: the Strong Heart Study (SHS) Am Heart J 2006 151 412 8 10.1016/j.ahj.2005.04.031 16442908

[B10] Nistri S Olivotto I Betocchi S Losi MA Valsecchi G Pinamonti B Conte MR Casazza F Galderisi M Maron BJ Cecchi F on behalf of Participating Centers. Prognostic significance of left atrial size in patients with hypertrophic cardiomyopathy (from the Italian Registry for Hypertrophic Cardiomyopathy) Am J Cardiol 2006 98 960 5 10.1016/j.amjcard.2006.05.013 16996883

[B11] Reed D Abbott RD Smucker ML Kaul S Prediction of outcome after mitral-valve replacement in patients with symptomatic chronic mitral regurgitation. The importance of left atrial size Circulation 1991 84 23 34 2060099 10.1161/01.cir.84.1.23

[B12] Gerdts E Wachtell K Omvik P Otterstad JE Oikarinen L Boman K Dahlof B Devereux RB Left atrial size and risk of major cardiovascular events during antihypertensive treatment: losartan intervention for endpoint reduction in hypertension trial Hypertension 2007 49 311 6 10.1161/01.HYP.0000254322.96189.85 17178978

[B13] Laukkanen JA Kurl S Eranen J Huttunen M Salonen JT Left atrium size and the risk of cardiovascular death in middle-aged men Arch Intern Med 2005 165 1788 93 10.1001/archinte.165.15.1788 16087829

[B14] Benjamin EJ D'Agostino RB Belanger AJ Wolf PA Levy D Left atrial size and the risk of stroke and death: the Framingham Heart Study Circulation 1995 92 835 41 7641364 10.1161/01.cir.92.4.835

[B15] Manning WJ Gelfand EV Left atrial size and postoperative atrial fibrillation: the volume of evidence suggests it is time to break an old habit J Am Coll Cardiol 2006 48 787 9 10.1016/j.jacc.2006.05.036 16904550

[B16] Helms AS West JJ Patel A Lipinki MJ Mangrum JM Mounsey JP Dimarco JP Ferguson JD Relation of left atrial volume from three-dimensional computed tomography to atrial fibrillation recurrence following ablation Am J Cardiol 2009 103 989 93 10.1016/j.amjcard.2008.12.021 19327428

[B17] Van Gelder IC VanGilst WH Verwer R Lie KI Prediction of uneventful cardioversion and maintenance of sinus rhythm from direct-current electrical cardioversion of chronic atrial fibrillation and flutter Am J Cardiol 1991 68 41 6 10.1016/0002-9149(91)90707-R 2058558

[B18] Tsang TS Abhayaratna WP Barnes ME Miyasaka Y Gersh BJ Bailey KR Cha SS Seward JB Prediction of cardiovascular outcomes with left atrial size: is volume superior to area or diameter? J Am Coll Cardiol 2006 47 1018 23 10.1016/j.jacc.2005.08.077 16516087

[B19] Bellenger NG Pennell DJ Manning WJ, Pennell DJ Ventricular function Cardiovascular magnetic resonance 2002 Churchill Livingstone, New York, USA

[B20] Grothues F Smith GC Moon JCC Bellenger NG Collins P Klein HU Pennell DJ Comparison of interstudy reproducibility of cardiovascular magnetic resonance with two-dimensional echocardiography in normal subjects and in patients with heart failure or left ventricular hypertrophy Am J Cardiol 2002 90 29 34 10.1016/S0002-9149(02)02381-0 12088775

[B21] Maceira AM Prasad SK Khan M Pennell DJ Normalized left ventricular systolic and diastolic function by steady state free precession cardiovascular magnetic resonance J Cardiovasc Magn Reson 2006 8 417 26 10.1080/10976640600572889 16755827

[B22] Maceira AM Prasad SK Khan M Pennell DJ Reference right ventricular systolic and diastolic function normalized to age, gender and body surface area from steady-state free precession cardiovascular magnetic resonance Eur Heart J 2006 27 2879 88 10.1093/eurheartj/ehl336 17088316

[B23] Moon JCC Lorenz CH Francis JM Smith GC Pennell DJ Breath-hold FLASH and FISP cardiovascular MR imaging: left ventricular volume differences and reproducibility Radiology 2002 223 789 97 10.1148/radiol.2233011181 12034951

[B24] Mosteller RD Simplified calculation of body surface area N Engl J Med 1987 317 1098 3657876 10.1056/NEJM198710223171717

[B25] Joint British Cardiac Society, British Hyperlipidaemia Association and British Hypertension Society recommendations on coronary prevention Heart 1998 80 Suppl 2 S1 29 PMC1766497 10193438

[B26] Maisel AS Krishnaswamy P Nowak RM McCord J Hollander JE Duc P Omland T Storrow AB Abraham WT Wu AH Clopton P Steg PG Westheim A Knudsen CW Perez A Kazanegra R Herrmann HC McCullough PA Breathing Not Properly Multinational Study Investigators. Rapid measurement of b-type natriuretic peptide in the emergency diagnosis of heart failure N Engl J Med 2002 347 161 7 10.1056/NEJMoa020233 12124404

[B27] Ujino K Barnes ME Cha SS Langins AP Bailey KR Seward JB Tsang TS Two-dimensional echocardiographic methods for assessment of left atrial volume Am J Cardiol 2006 98 1185 8 10.1016/j.amjcard.2006.05.040 17056324

[B28] Hudsmith LE Petersen SE Francis JM Robson M Neubauer S Normal human left and right ventricualr and left atrial dimensions using steady state free precession magnetic resonance imaging J Cardiovasc Magn Reson 2005 7 775 82 10.1080/10976640500295516 16353438

[B29] Sievers B Kirchberg S Franken U Bakan A Addo M John-Puthenveettil B Trappe HJ Determination of normal gender-specific left atrial dimensions by cardiovascular magnetic resonance imaging J Cardiovasc Magn Reson 2005 7 677 83 16136858 10.1081/jcmr-65621

[B30] Sievers B Addo M Kirchberg S Bakan A John-Puthenveettil B Franken U Trappe HJ How much are atrial volumes and ejection fractions assessed by cardiac magnetic resonance imaging influenced by the ECG gating method? J Cardiovasc Magn Reson 2005 7 587 93 10.1081/JCMR-200060635 15959972

[B31] Anderson JL Horne BD Pennell DJ Atrial dimensions in health and left ventricular disease using cardiovascular magnetic resonance J Cardiovasc Magn Reson 2005 7 671 5 16136857 10.1081/jcmr-65617

[B32] Therkelsen SK Groenning BA Svendsen JH Jensen GB Atrial and ventricular volume and function in persistent and permanent atrial fibrillation, a magnetic resonance imaging study J Cardiovasc Magn Reson 2005 7 465 73 10.1081/JCMR-200053618 15881530

[B33] Nikitin NP Witte KK Tackray SD Goodge LJ Clark AL Cleland JG Effect of age and sex on left atrial morphology and function Eur J Echocardiogr 2003 4 36 42 10.1053/euje.2002.0611 12565061

[B34] Tseng WY Liao TY Wang JL Normal systolic and diastolic functions of the left ventricle and left atrium by cine magnetic resonance imaging J Cardiovasc Magn Reson 2002 4 443 57 10.1081/JCMR-120016383 12549232

[B35] Thomas L Levett K Boyd A Leung DY Schiller NB Ross DL Compensatory changes in atrial volumes with normal aging: is atrial enlargement inevitable? J Am Coll Cardiol 2002 40 1630 5 10.1016/S0735-1097(02)02371-9 12427416

[B36] Germans T Götte M Nijveldt R Spreeuwenberg MD Beek AM Bronzwaer JGF Visser CA Paulus WJ van Rossum AC Effects of aging on left atrioventricular coupling and left ventricular filling assessed using cardiac magnetic resonance imaging in healthy subjects Am J Cardiol 2007 100 122 7 10.1016/j.amjcard.2007.02.060 17599453

[B37] Ujino K Barnes ME Cha SS Langins AP Bailey KR Seward JB Tsang TSM Two-dmensional echocardiographic methods for assessment of left atrial volume Am J Cardiol 2006 98 1185 8 10.1016/j.amjcard.2006.05.040 17056324

[B38] Keller Am Gopal AS King DL Left and right atrial volume by freehand three-dimensional echocardiography: in vivo validation using magnetic resonance imaging Eur J Echocardiogr 2000 1 55 65 10.1053/euje.2000.0010 12086217

[B39] Artang R Migrino RQ Harmann L Bowers M Woods TD Left atrial volume measurement with automated border detection by 3-dimensional echcoardiography: comparison with magnetic resonance imaging Cardiovasc Ultrasound 2009 7 16 24 10.1186/1476-7120-7-16 19335908 PMC2669050

[B40] Lin FY Devereux RB Roman MJ Meng J Jow VM Jacobs A Weinsaft JW Shaw LJ Berman DS Callister TQ Min JK Cardiac chamber volumes, function and mass as determined by 64-multidetector row computed tomography: mean values among healthy adults free of hypertension and obesity JACC Cardiovasc Imaging 2008 1 782 6 10.1016/j.jcmg.2008.04.015 19356515

[B41] Mahabadi AA Samy B Seneviratne SK Toepker MH Bamberg F Hoffmann U Truong QA Quantitative assessment of left atrial volume by electrocardiographic-gated contrast-enhanced multidetector computed tomography J Cardiovasc Comput Tomogr 2009 3 80 7 10.1016/j.jcct.2009.02.002 19332340 PMC2672427

[B42] Sievers B Kirchberg S Addo M Bakan A Brandts B Trappe HJ Assessment of left atrial volumen in sinus rythm and atrial fibrillation using the biplano area-length method and cardiovascular magnetic resonance imaging with TrueFISP J Cardiovasc Magn Reson 2004 6 855 63 10.1081/JCMR-200036170 15646889

[B43] Badano LP Pezzutto N Marinigh R Cinello M Nucifora G Pavón D Gianfagna P Fioretti PM How many patients would be misclassified using M-mode two-dimensional estimates of left atrial size instead of left volume? A three-dimensional echocardiographic study J Cardiovasc Med (Hagerstown) 2008 9 476 84 10.2459/JCM.0b013e3282f194f0 18403999

[B44] Rodevan O Biornerheim R Ljosland M Ámele J Smith HJ Ihien H Left atrial volumes assessed by three- and two-dimensional echocardiography compared to MRI estimates Int J Card Imaging 1999 15 397 410 10.1023/A:1006276513186 10595406

